# Shape-Sensing of Beam Elements Undergoing Material Nonlinearities

**DOI:** 10.3390/s21020528

**Published:** 2021-01-13

**Authors:** Pierclaudio Savino, Marco Gherlone, Francesco Tondolo, Rita Greco

**Affiliations:** 1 Department of Structural, Geotechnical and Building Engineering, Politecnico di Torino, Corso Duca degli Abruzzi 24, 10129 Torino, Italy; francesco.tondolo@polito.it; 2 Department of Mechanical and Aerospace Engineering, Politecnico di Torino, Corso Duca degli Abruzzi 24, 10129 Torino, Italy; marco.gherlone@polito.it; 3 Department of Civil Engineering, Environmental, Territory, Building and Chemical, Politecnico di Bari, Via Edoardo Orabona 4, 70125 Bari, Italy; rita.greco@poliba.it

**Keywords:** iFEM, nonlinearities, strain monitoring, structural health monitoring, displacements

## Abstract

The use of in situ strain measurements to reconstruct the deformed shape of structures is a key technology for real-time monitoring. A particularly promising, versatile and computationally efficient method is the inverse finite element method (iFEM), which can be used to reconstruct the displacement field of beam elements, plate and shell structures from some discrete strain measurements. The iFEM does not require the knowledge of the material properties. Nevertheless, it has always been applied to structures with linear material constitutive behavior. In the present work, advances are proposed to use the method also for concrete structures in civil engineering field such as bridges normally characterized by material nonlinearities due to the behavior of both steel and concrete. The effectiveness of iFEM, for simply supported reinforced concrete beam and continuous beams with load conditions that determine the yielding of reinforcing steel, is studied. In order to assess the influence on displacements and strains reconstructions, different measurement stations and mesh configurations are considered. Hybrid procedures employing iFEM analysis supported by bending moment-curvature relationship are proposed in case of lack of input data in plastic zones. The reliability of the results obtained is tested and commented on to highlight the effectiveness of the approach.

## 1. Introduction

The efficiency of civil structures has a crucial role, with economic and social impacts. On the other hand, processes like corrosion, fatigue, erosion and overloads worsen structures’ behavior, making them no longer suitable for the intended use according to the latest safety requirements. For these reasons, the employment of appropriate structural health monitoring (SHM) strategies has strategic importance and provides a continuous condition assessment, overviews the safety level and optimizes maintenance operations reducing cost and downtime. Despite this great potential and the current advancements in the sensor research field, SHM is not yet widely applied in a systematic manner to civil structures and infrastructures [1]. Among the main reasons there are the complexity and cost of monitoring large-scale constructions, the processing of huge amount of data and the difficulty of inspection. One technique that has relevant implications in structural health monitoring and that seems to overcome some of these challenges is “shape-sensing”. The key capability of this methodology is the real-time evaluation of the structural response in complex structures with the use of few strain sensors placed in easily accessible points. The knowledge of the full-field displacements implies that further structural response, such as the stress field, can be reconstructed, thus enabling real-time damage prediction by means of proper failure criteria. Furthermore, the advances in the microelectromechanical system (MEMS) field have made this possible with low-cost devices [2,3]. Embedded systems are also welcome because they can be protected into the structure against the aggressive external environment that causes failures in traditional external systems.

Existing shape-sensing methods are based on numerical integration of experimental strains, on continuous basis functions that approximate the displacement field, on the use of neural networks or variational principles. A great effort for the predictions of beam deflection integrating discretely measured strains has been performed by Ko et al. [4]. The displacement theory developed by Ko et al. [4], formulated for the predictions of cantilevered beam deformed shapes, involved the integration of the differential equations for uniform section beam. For the full-field reconstruction of more complex deformed shapes, some authors introduced an a priori set of global or piecewise continuous basis functions to fit measured strains. Kang et al. [5] used a mode-shape approach to obtain a displacement–strain relationship. Multiplying measured strain data, and the displacement–strain relationship matrix, the beam response in terms of displacements can be reconstructed. A different class of methods involves neural networks for the shape reconstruction of frame structures. Bruno et al. [6] proposed a neural network model with two fully connected layers, where the input layer represents the measurements of length changes of the structural members, and the output layer contains the total number of unknown DOF in the system. However, most of the methods belonging to these classes require loading and/or material information, the use of the global equilibrium conditions and the result to be not enough general.

An algorithm that allows the shape-sensing for various structural typologies and boundary conditions in a robust and stable way and enough fast for real-time applications is the inverse finite element method (iFEM). The method, developed by Tessler and Spangler [7,8] for shear-deformable plate and shell structures based on Mindlin theory, employs a variational principle based on a least-square functional discretized by C^0^-continuous finite elements. Minimization of the least-square functional with respect to nodal degrees of freedom leads to enforcing the compatibility between the measured and interpolated strains for each inverse element. After the introduction of this first iFEM-based element, Kefal et al. [9] proposed a four-node quadrilateral inverse-shell element considering Mindlin theory as a kinematic framework. Kefal [10] developed a new eight-node curved inverse-shell element named iCS8. The kinematic relations are obtained by combining the kinematics assumptions of Mindlin plate theory and of a solid shell. Most recently, Kefal and Oterkus [11] presented an isogeometric iFEM methodology for shape-sensing analyses of complex thin plate and shell structures. Zhao et al. [12] proposed a new iFEM formulation for reconstructing the displacement field of variable cross-section beams based on isogeometric analysis. The iFEM was extended by Gherlone et al. [13,14] for the shape-sensing of truss, beam and frame structures based on Timoshenko beam theory. Two inverse finite elements were introduced with 0th order and 1st order approximation, respectively. Some applications of the iFEM to cantilever beams and three-dimensional frame structures were presented. The iFEM formulation was extended by Savino et al. [15] to beam and frame structures based on the kinematical assumptions of the Bernoulli–Euler theory. Two inverse finite elements were formulated, 0th and 1st order, for both concentrated and distributed load conditions, respectively. The most common structural types of the civil engineering field were discussed, such as cantilevered, continuous beam and frame structures. In order to increase the use of the iFEM formulation, also in the case of curved beams, Savino et al. [16] developed a new two-node curved inverse-beam element by utilizing the kinematic assumptions of Bernoulli–Euler curved beam theory. The studies performed on beam elements type [13,14,15,16] highlighted the efficiency in predicting the structural responses in the case of linear elastic behavior.

A large-displacement analysis using the shell iFEM theory has been performed by Tessler et al. [17]. An incremental procedure is carried out to take into account the geometric nonlinearities, where at each load step, the measured incremental strains are used to evaluate the incremental displacements, which in turn update the geometry of the deformed shape. The predictive capability of the iFEM formulation is also demonstrated using experimentally measured strains in complex aerospace structures (Quach et al. [18], Vazquez et al. [19], Cerracchio et al. [20]), marine structures (Kefal and Oterkus [21], Kefal and Oterkus [22], Kefal and Oterkus [23]) and offshore wind turbine towers (Li et al. [24]).

The main aim of this work is to verify, for the first time in the open literature, the effectiveness of iFEM in the case of both statically determinate and indeterminate structures with material nonlinearities. In particular, simply supported and continuous beams are studied because they represent a very common static scheme in civil engineering for bridges. None of the numerical or experimental shape and strain sensing applications presented so far consider the material nonlinearities of a reinforced concrete beam. To this purpose, the formulation of the iFEM algorithm using the Bernoulli–Euler inverse element described in Savino et al. [15] has been adopted. First of all, some numerical examples have been considered to study the influences of the number of strain sensors (station points) and mesh discretization in the iFEM framework. A relevant difference is found compared to the iFEM application on structures with linear-elastic behavior due to the high dependence of the results both on the location of the sensor and on the mesh refinement. Consequently, some changes are proposed for both modeling and choice of the station points location. Furthermore, alternative procedures have been developed for statically determinate and indeterminate structures to use iFEM analysis even in the case of lack of station points in the zones of the structures where nonlinear material behavior is registered. The introduction of the bending moment-curvature diagram of the section property as a tool in the procedure has added further potential for smart monitoring of structures. Direct nonlinear FEM analyses using ADINA software are performed both to simulate the experimental strain measurements in discrete points and also to establish the reference solution for displacements and curvatures along the structure; the strain measurements are then used by iFEM to reconstruct the deformed shape. Finally, the comparison between reference solutions and iFEM predictions, appropriately extended to the case of nonlinear material behavior, demonstrated the effectiveness of the procedure proposed to reconstruct both displacement and strain fields for reinforced concrete. The knowledge of the deformed shape could be used from the practical point of view to monitor the displacements, comparing them to alert thresholds. Furthermore, by introducing constitutive equations, stress-sensing and then real-time damage prediction could be enabled.

## 2. Structural Behavior of Reinforced Concrete Beams

**Figure 1:**
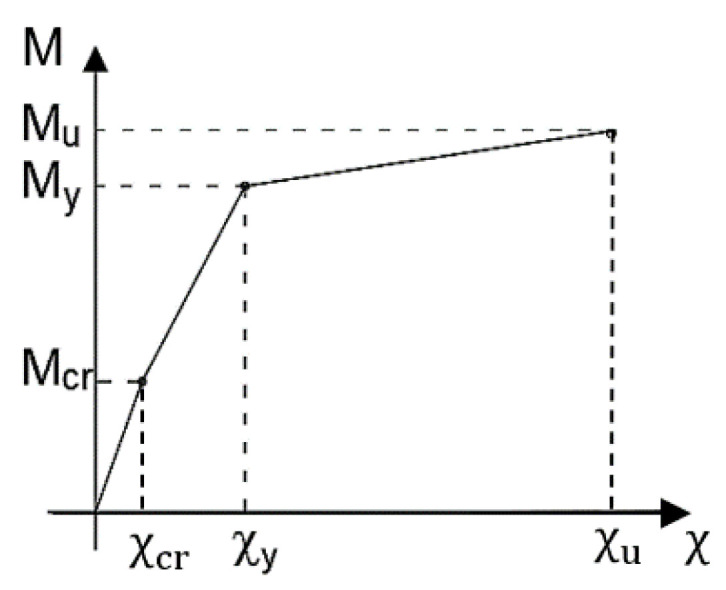
Ideal bending-moment curvature diagram.

In reinforced concrete beam elements, there are several sources for nonlinear behavior; the most common is due to the cracking of concrete that appears when the tensile stress in the concrete overcomes the tensile strength. In this case, the cross-section is no longer entirely reactive, and consequently, the structural behavior is nonlinear. For beams, the nonlinear behavior can be taken into account with the bending moment-curvature diagram that describes the structural response of a reinforced concrete section in bending, assuming a planar deformation. In particular, the bending moment-curvature diagram can be obtained from the geometry and the stress–strain relationships of the materials of the sections by assuming a perfect reinforcing steel-concrete bond and no axial actions applied. The diagram is schematically characterized by three main branches (Figure 1):

- First uncracked elastic phase, with a pattern close to the linear and curvature ranging from 0 to χ_cr_. The tensed concrete contributes to the resistance of the structure up to the cracking bending moment (M_cr_), where stress in concrete in tension exceeds its tensile strength;
- Second phase with cracked section, bending moment ranging from the cracking bending moment (M_cr_) and the reinforcement yielding bending moment (M_y_), increasing levels of curvature and higher deformability;
- Third phase with yielded reinforcements, showing a rapid increase of the curvature pattern until the ultimate bending moment (M_u_) is reached when one of the two materials, reinforcing steel or concrete, experiences the maximum nominal strain.

In the case of statically determinate structures, the nonlinearities affect only the deformation level because the actions are not depending on the stiffness of the elements, and they are defined by the static equilibrium equations. For statically indeterminate structures, in addition to a change in the deformation state, there is also a redistribution of stresses that are no longer linear with the increasing loads. Due to the reduction of stiffness with the occurrence of cracks, the bending moment increases more rapidly in areas that are still in the elastic phase. For higher loads, the stresses increase according to the pattern of the cracks until the yielding strength of the reinforcement is reached. This corresponds to the formation of plastic hinges that allow rotations with a very slight increase in bending moment. To ensure balance, the bending moment must grow faster in the sections in the second phase until reaching the yielding of steel. The collapse occurs when all the ductility reserve is lost in the first section.

## 3. Basics of iFEM

In this section, a brief description of the iFEM procedure for beam structures is presented. Depending on the particular structural theory (Timoshenko, Bernoulli–Euler), the displacement field can be written in terms of some kinematic variables, u(z), varying along the axial beam coordinate, z. The vector of kinematic variables u(z) can be interpolated within each inverse finite element by appropriate shape functions:

(1) $$u\left(z\right)=N\left(z\right)\cdot u^{e}$$

where N(z) indicates the shape functions matrix and u^e^ denotes the nodal degrees of freedom. According to the kinematic assumptions of the theory [7,8,9,13,14,15,16], the corresponding strain field e(z) can be expressed as a function of the nodal degrees of freedom:

(2) $$e\left(z\right)=B\left(z\right)\cdot u^{e}$$

where B(z) is the matrix of the shape functions of the strains. To reconstruct the deformed shape by in situ strain measurements, the iFEM is based on a functional representing the least-squares error between the analytic section strains e(u) and the corresponding experimental section strains e^ε^:

(3) $$\Phi \left(u\right)=||e\left(u\right)-e^{\varepsilon }||{}^{2}$$

Thus, in the case of discretization with m elements, the total least-square functional is a sum of the individual element contributions:

(4) $$\Phi =\overset{m}{\underset{e=1}{\sum }}\Phi^{e}$$

where the error functional Φ^e^ is defined as the sum of the products of the weighting coefficient vector w_k_ and of the least-squares component vector $\Phi_{k}^{e}$ for each kth independent strain:

(5) $$\Phi^{e}=w_{k}\cdot \Phi_{k}^{e}$$

The weighting coefficients contain dimensional parameters and dimensionless coefficients that enforce a stronger or weaker correlation between the analytic and experimentally measured counterparts. The functional $\Phi_{k}^{e}$ is defined as:

(6) $$\Phi_{k}^{e}=\frac{1}{n}\overset{n}{\underset{i=1}{\sum }}{\left[e_{k}\left(z_{i}\right)-e_{k}^{\varepsilon i}\right]}^{2}$$

where n is the number of station points (z_i_) within the element, i.e., locations where the strains are measured. The minimization of the functional (5) with respect to the nodal degrees of freedom leads to the inverse element matrix equation:

(7) $$\left[S^{e}\right]\cdot \left\{u^{e}\right\}=\left\{h^{e}\right\}$$

where the matrix [$S^{e}$] depends only on the strain sensors locations, whereas the vector {h^e^} is also a function of the experimental strain measurements. Applying the coordinate transformation from the element-local to the structure-global reference system, assembling the contribution of each element and enforcing the boundary conditions, the non-singular global equations in matrix form are obtained:

(8) [S]·{u}={h}

By inverting the matrix [S], the system can be solved for the unknown degrees of freedom and the deformed shape of the whole structure can be reconstructed. It is important to observe that, since the matrix [S] remains unchanged for a given distribution of strain sensors and is independent of the measured strain values, it can be inverted only once also for dynamic applications, thus leading to a computationally efficient algorithm also for real-time monitoring.

The remaining part of the formulation involves the selection of suitable shape functions according to the number of required strain sensors and the evaluation of the experimental section strains. In the present work, since numerical simulations of structures subjected to uniformly distributed load are performed, the 1st order inverse element developed by Savino et al. [15] has been adopted. This inverse element is based on the kinematic assumptions of the Bernoulli–Euler beam theory, using 4th degree Hermitian shape functions with two nodes and twelve degrees of freedom. Refer to Savino et al. [15] for further details on the formulation.

## 4. iFEM Algorithm with Material Nonlinearity

Since the iFEM was initially created to determine the elastic structural response of aerospace vehicles using in-flight strain measurements, all the works previously carried out to test the effectiveness of the method concerns experimental tests or numerical simulations of homogeneous aluminum elements [18,19,21,22,23,25,26,27] or thin-walled multilayered composite structures [20,28]. In the present work, the applicability of the method for statically determinate and indeterminate reinforced concrete beams with nonlinear material behavior is studied by performing numerical simulations. Nonlinear direct FEM analyses are used to provide both the strain measurements as input data for the following iFEM analysis and the displacements as reference results for those predicted via iFEM. The input strains considered as the starting point of the iFEM procedure are related to the curvature, and axial strain referred to the centroidal axes. The beam cross-section is modeled using the bending moment-curvature material model in ADINA software. The direct FEM analyses are carried out using highly refined beam element models (30 elements). The used ADINA element is a 2-node 3rd degree Hermitian beam with two nodes and twelve degrees of freedom. The accuracy of the iFEM prediction is assessed by evaluating both the root-mean-square (RMS) difference and the percentage difference between the iFEM-predicted quantities and those evaluated with the direct ADINA FE model:

(9) $${\%e}_{\mathrm{RMS},x}=100\cdot \sqrt{\frac{1}{p}\overset{p}{\underset{i=1}{\sum }}{\left(\frac{x_{i}^{\mathrm{iFEM}}-x_{i}^{\mathrm{FEM}}}{x_{\max}^{\mathrm{FEM}}}\right)}^{2}}$$

(10) $${\%e}_{\mathrm{Diff},x}=100\cdot \frac{x_{i}^{\mathrm{iFEM}}-x_{i}^{\mathrm{FEM}}}{x_{\max}^{\mathrm{FEM}}}$$

where “x” indicates the compared quantity (displacements e_Diff,d_ and curvatures e_Diff,c_) and “p” is the number of nodes.

### 4.1. Statically Determinate Structures

**Figure 2:**
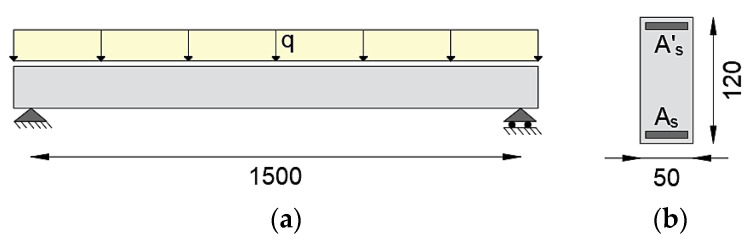
(**a**) Static scheme of the statically determinate beam; (**b**) cross-section geometry (dimensions in cm).

The application of the iFEM with material nonlinearity in a statically determinate element is studied considering a simply supported beam with a span of 15 m, subjected to a distributed load q (Figure 2a). The cross-section has a base of 0.5 m and height of 1.2 m, is doubly reinforced with ordinary reinforcing steel for concrete structures with yielding stress of 450 MPa, ultimate stress equal to 510 MPa, the elastic modulus of 200 GPa, resisting areas As = 9074.60 mm^2^ in the tensed zone and As’ = 3532.50 mm^2^ in the compressed zone with a concrete cover of 5 cm (Figure 2b). It is assumed that the beam has the same amount of reinforcement along the entire length. The concrete compressive strength has been considered of 30 MPa, corresponding to an elastic modulus of 32 GPa.

**Figure 3:**
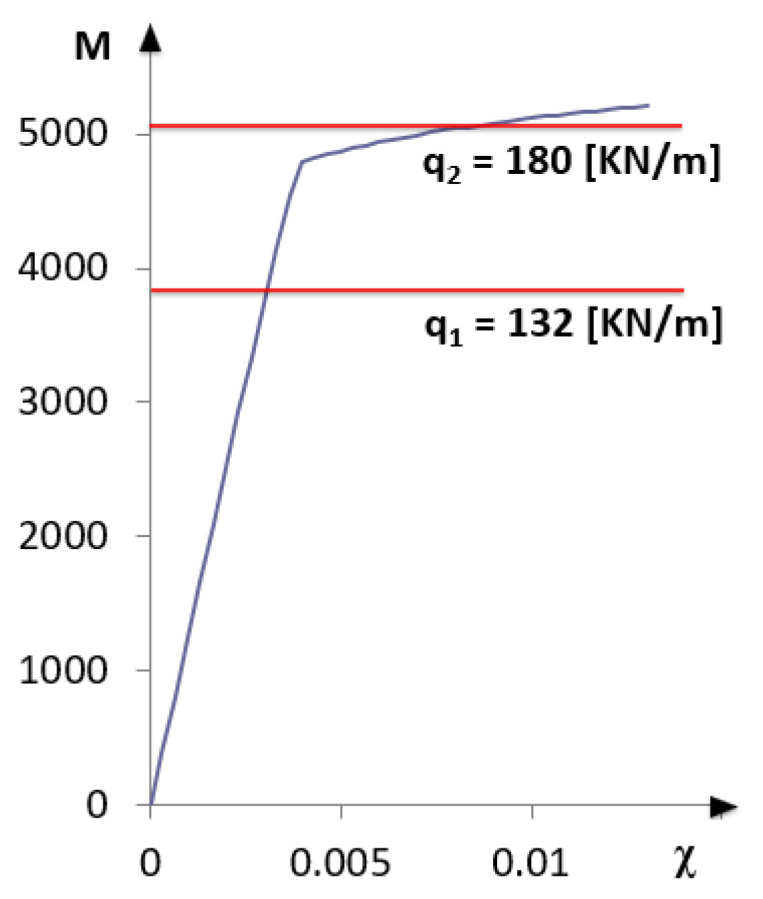
Bending moment-curvature diagram with load level.

Following the procedure briefly described in Section 2, it is possible to obtain the bending moment-curvature reported in Figure 3.

As a first step, a uniformly distributed load q_1_ = 132 KN/m is considered, which produces in the midspan of the simply supported beam a maximum bending moment of about 80% of the yielding bending moment (M_y_ = 4790 KN·m). The reinforcing bars are, therefore, working within the elastic regime (Figure 3).

**Figure 4:**
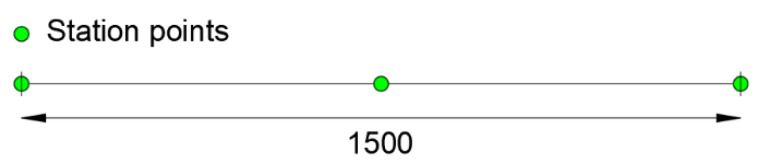
1st order element with three station points.

For the iFEM analysis, the beam meshes with only one 1st order element [15] with three station points at z_i_ = 0, 7.5 and 15 m. Figure 4 shows a scheme of the inverse element used to model the beam, where the positions of the station points are depicted with green dots, and the extremities of the inverse element are denoted with vertical black marks.

**Figure 5:**
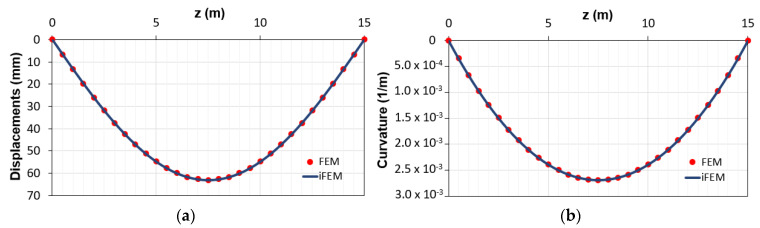
Comparison between direct finite element method (FEM) and inverse finite element method (iFEM) solutions in terms of (**a**) displacements diagram; (**b**) curvature diagrams.

The comparisons between both the vertical displacements and the corresponding curvatures obtained using the direct FEM (red points) and iFEM (blue line) analyses are reported in Figure 5. According to the previous works with linear elastic elements [14,15], the two sets of outputs are in good agreement. These results are due to the small nonlinearities present along the element given by the concrete cracking in a limited portion across the beam, which is characterized by an average behavior between the 1st and 2nd phase. Indeed, from Figure 5, the difference between cracked and uncracked stage (1st and 2nd phases, respectively) is not visible due to the structural response of such a kind of reinforced concrete beam. The RMS percent error is 0.053% for the displacements and 0.036% for the curvatures.

**Figure 6:**
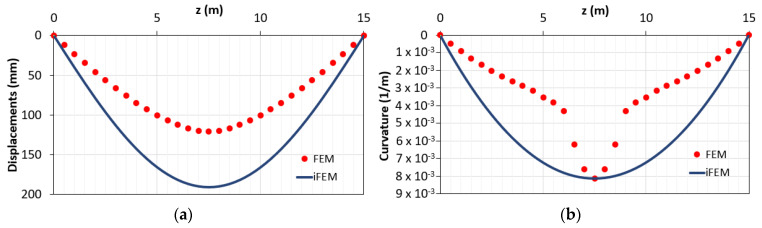
Yielded element modeled with one inverse element and three station points: (**a**) displacements diagram; (**b**) curvatures diagrams.

Increasing the load up to q_2_ = 180 KN/m, a maximum bending moment of approximately 97% of the ultimate bending moment (M_u_ = 5208 KN·m) is produced (Figure 3). In this case, in a zone of about 3 m across the midspan of the beam, the acting bending moment exceeds the yielding bending moment, as reported in Figure 6.

As in the first modeling, the same configuration of the previous example is considered (Figure 4). In Figure 6, the comparisons of the displacements and curvatures diagrams obtained with FEM and iFEM analyses are reported. It can be seen that considering two input curvatures from zones in the 1st–2nd phases (support) and another from the zone in the 3rd phase (mid-span), displacements and curvatures are overestimated by iFEM.

Unlike the linear-elastic case, in the present analysis, two aspects should be considered:

- not all the station points belong to zones with the same behavior (1st–2nd or yielding phase);
- each span with a different behavior needs to be modeled with a different inverse element.

**Figure 7:**
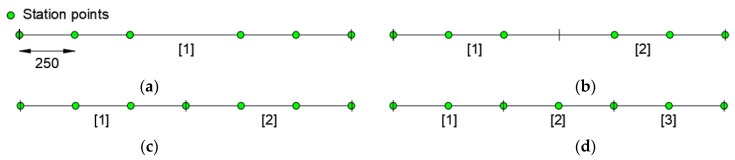
Modeling configuration: (**a**) one element with six station points; (**b**) two elements with three station points; (**c**) two elements with four station points; (**d**) three elements with three station points.

Taking into account the two observations above, an increase of station points and mesh discretization is investigated (Figure 7). In particular, configurations “a” and “b”, with one and two elements, respectively, have no station points in the yielded zone (Figure 7a,b), whereas configurations “c” and “d”, with two and three elements, respectively, have station points located in the yielded zone (Figure 7c,d). Configurations “a” and “b”, have been obtained with inverse elements having, respectively, three and one redundant station points with respect to the strictly required [15].

**Figure 8:**
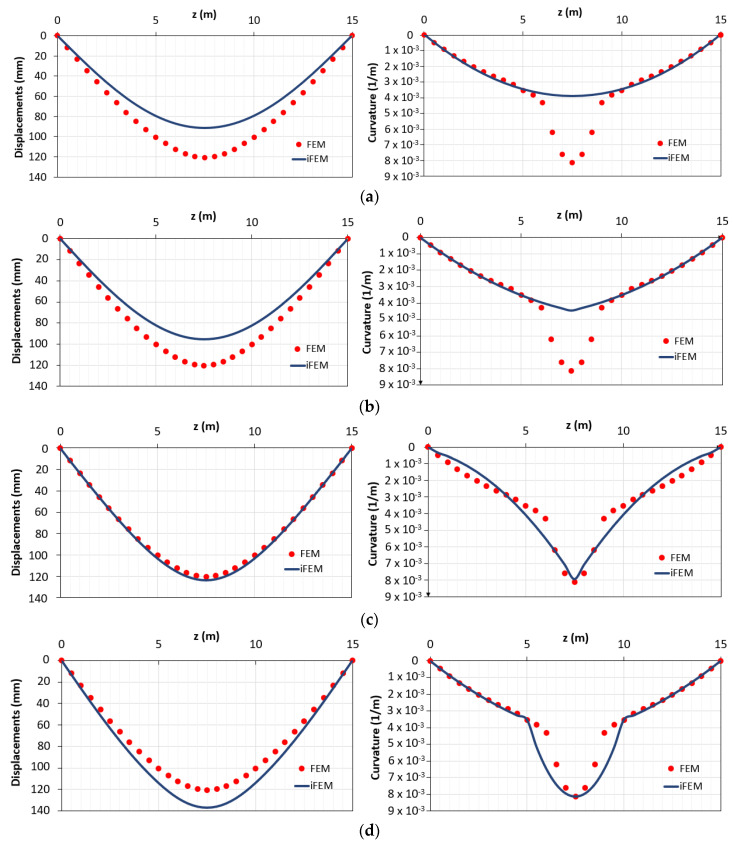
Displacements and curvatures diagrams for: (**a**) configuration “a”; (**b**) configuration “b”; (**c**) configuration “c”; (**d**) configuration “d”.

Figure 8 shows the results for the configurations “a”, “b”, “c” and “d”.

In Table 1, the errors for the different configurations are reported.

**Figure 9:**
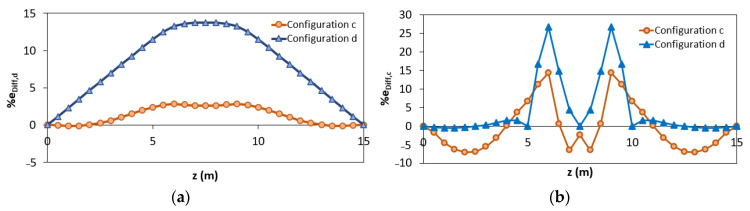
Percentage difference using configurations “c” and “d”: (**a**) iFEM-predicted displacements; (**b**) iFEM-predicted curvature.

Comparing the results of the configuration “a” and “b”, it can be seen that better results are obtained considering two elements instead of one. Nevertheless, the best results are obtained when the station points are also placed in the yielded area (configuration “c” and “d”). To better clarify the results obtained according to the different configurations “c” and “d”, it is possible to analyze the percentage difference e_Diff,x_ along the length of the structure (Figure 9a,b).

It can be seen that, unlike in configuration “c” (where each inverse element interpolates discontinuous curvatures within the three different phases), for configuration “d” there are inaccuracies mainly in the central element (between z = 5 m and z = 10 m) because it includes both zone in 2nd and 3rd phase (Figure 9b). Due to this reason, the displacements of the configuration “d” are overestimated (Figure 9a). Therefore, configuration “d” can be considered as the most accurate in terms of reconstructed curvature because it shows a lower percentage difference of the curvature for a longer length despite the overestimation along the plastic zone.

#### 4.1.1. Hybrid Method for Statically Determinate Structures

The results described above show some critical issues: the need to estimate the curvatures in the yielded zones when not instrumented (“a” and “b” configurations), the request to properly model the yielded zone with an element to avoid overestimation of the curvatures (“c” and “d” configurations). Indeed, the iFEM method should be integrated by some data from the mechanical model, referring to its structural characteristics and curvature trends. At the same time, it should be specified that the lack of measurements in the middle span is the most disadvantageous case and also the least probable given the importance of controlling this zone in statically determinate beams. Furthermore, it is possible to exploit the characteristics of the simply supported structure in terms of bending moment distribution. Therefore, in order to improve the performance of the method, the following hybrid procedure can be proposed.

In case of missing input data in the plastic zone (configurations “a” and “b”), one can:

1. Find the initial curvature measurement (from the strain measurements) and using the bending moment-curvature diagram, get the corresponding bending moment value at the station points;
2. Reconstruct the bending moment diagram along the whole beam span with a polynomial function interpolating the values obtained in step (1). In the case of *l* estimated bending moment values, the polynomial function M(z) has the form:
  (11) $$M\left(z\right)=a_{1}+a_{2}z+\ldots +a_{l}z^{l-1}$$
3. Identify the beginning and the end of the plastic zone by comparing the values of the bending moment diagram and M_y_ (the plastic zone must correspond to an inverse element characterized by “auxiliary points”, i.e., additional station points);
4. Evaluate the curvatures and the bending moment values at the auxiliary points considering the bending moment-curvature diagram;
5. Perform iFEM analysis using the measured and estimated (auxiliary points) curvatures as input data.

It can be observed that the maximum deviation between the reconstructed bending moment diagram by the interpolating polynomial and the real one could occur in the case of the unloaded zone only in the middle span. However, this worst case is always avoided by the effect of the dead load of the beam.

In the case of input data also in the plastic area, the proposed procedure should be performed from step (1) to step (3) in order to identify the beginning and the end of the element where to place the auxiliary points to model the plastic zone.

**Figure 10:**
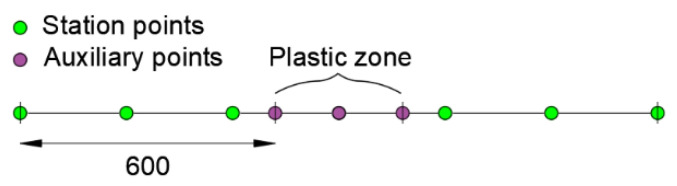
Modeling scheme for the proposed procedure.

Applying the method for the same station points considered in the configurations “a” and “b”, the following modeling scheme is adopted (Figure 10).

**Figure 11:**
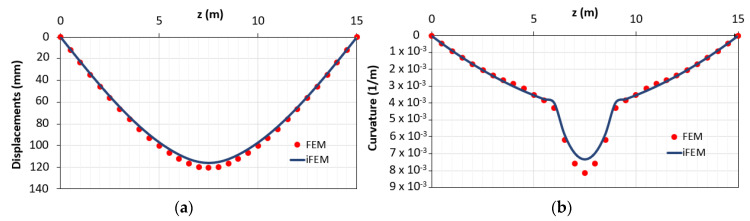
Results obtained with the proposed method in cases of lack of data in the plastic area in terms of (**a**) displacements; (**b**) curvature.

In Figure 11, the displacement and curvature diagrams obtained with the hybrid procedure are reported.

**Figure 12:**
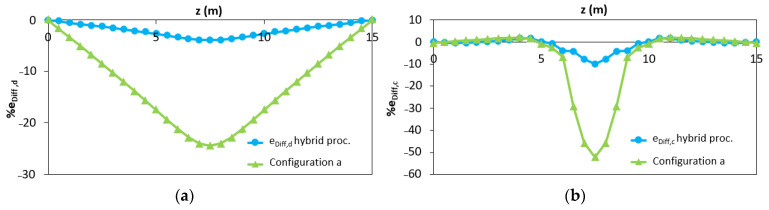
Distribution of the percentage difference in case of the hybrid procedure (blue curves) and without hybrid procedure (green curves) in (**a**) iFEM-predicted displacements; (**b**) iFEM-predicted curvature.

The proposed method, which allows applying the iFEM algorithm to reinforced concrete statically determinate elements in case of lack of data in the plastic area, is accurate both in terms of RMS displacements (2.31%) and RMS deformations (3.25%). In Figure 12, the distribution of the percentage differences along the beam in terms of displacements (e_Diff,d_) and curvatures (e_Diff,c_) obtained with (blue curves) and without (green curves) hybrid procedure is reported. The green curves refer to the outputs shown in Figure 8a.

**Figure 13:**
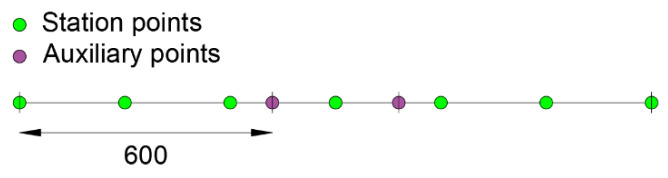
Modeling configuration in the procedure for estimating the plastic zone.

Finally, the procedure to identify the beginning and the end of the element where to model the plastic zone is applied, considering the station points of the configuration “c” and “d”. The same steps from (1) to (3) performed in the previous example are used, and then a similar configuration related to the auxiliary station points (beginning and end of the plastic zone) is considered. The difference with respect to the previous example is that the curvature in the central station point is a known input data (see green bullet between the two violet bullets in Figure 13).

**Figure 14:**
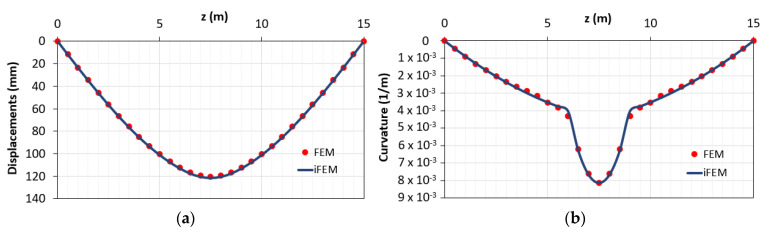
Output in case of the estimate of the central element length: (**a**) displacements diagram; (**b**) curvature diagram.

In Figure 14, the displacements and curvature diagrams are reported.

**Figure 15:**
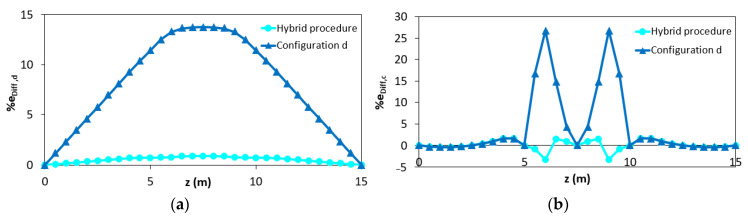
Distribution of the percentage difference in case of the hybrid procedure (blue curves) and without hybrid procedure (light blue curves) in (**a**) iFEM-predicted displacements; (**b**) iFEM-predicted curvature.

As expected, the results are even more accurate than in the previous example because the maximum curvature is input from the station points. In Figure 15, the comparison of the percentage differences with respect to that obtained considering the configuration “d” without the hybrid procedure is reported.

From the results shown in Figure 12 and Figure 15, it can be seen that the proposed hybrid method allows for obtaining more accurate results than the traditional iFEM procedure.

### 4.2. Statically Indeterminate Structures

**Figure 16:**
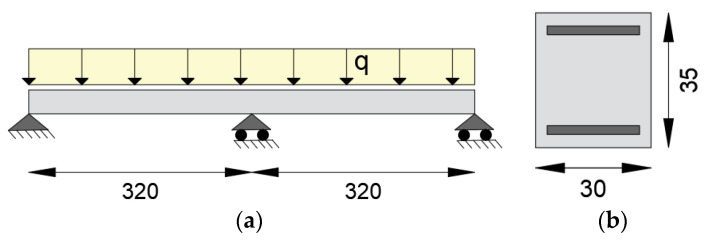
(**a**) Static scheme of the continuous beam; (**b**) cross-section geometry (dimensions in cm).

To test the applicability of the iFEM to statically indeterminate reinforced concrete structures, a continuous beam with two spans of 3.2 m and uniformly distributed load q is considered (Figure 16a). The rectangular cross-section of 0.30 × 0.35 m is symmetrically reinforced with As = As’ = 1256 mm^2^ (Figure 16b) and has a concrete cover of 35 mm. Both the concrete and the reinforcing steel characteristics are equal to the previous example. The beam is considered uniformly reinforced from the supports to the middle span.

**Figure 17:**
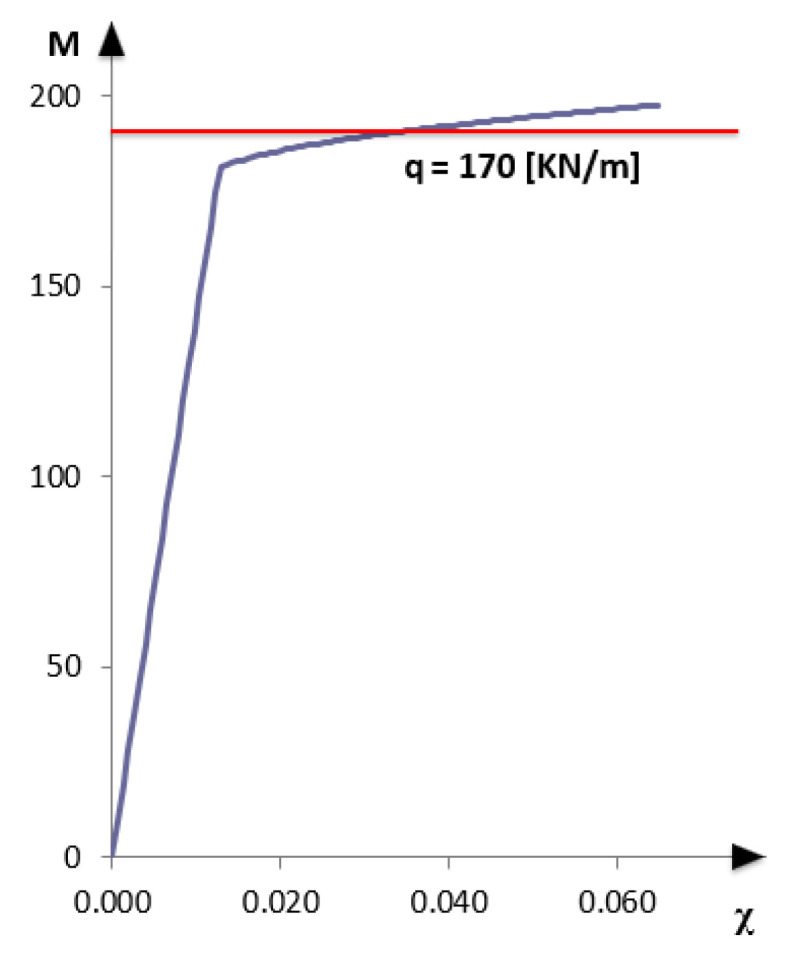
Bending moment-curvature diagram (q = 170 KN/m).

The statically indeterminate structure is represented in Figure 16. Since in this example the cracking stage is neglected, only two phases, namely before and after yielding of steel reinforcement, are represented in the bending-moment curvature diagram (Figure 17). The study of the nonlinear behavior and the applicability of the iFEM focuses on the phase in which the acting bending moment on the central support exceeds the yielding bending moment (M_y_), and a redistribution of the actions takes place. A load of 170 KN/m was considered that allows to reach on the central support a bending moment of about 97% of the ultimate bending moment (M_u_ = 197.76 KN·m) and therefore greater than the yielding bending moment.

If all the sections of the structure are in the 1st or 2nd phase, the observations made in the previous paragraph concerning a bending moment M < M_y_ are valid.

**Figure 18:**
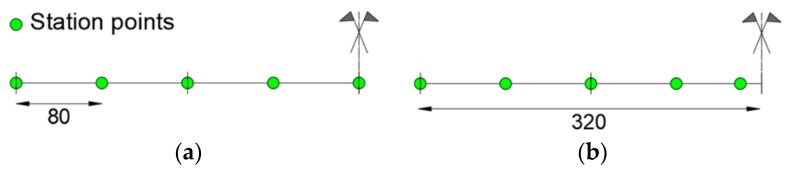
Modeling left-half of the structure configurations: (**a**) plasticized zone with station point; (**b**) plasticized zone without station point.

Two iFEM modeling configurations with (Figure 18a) and without (Figure 18b) station points in the plasticized zone are considered. Both the configurations are modeled with four inverse elements. Figure 18 shows only the left half of the structure due to symmetry.

**Figure 19:**
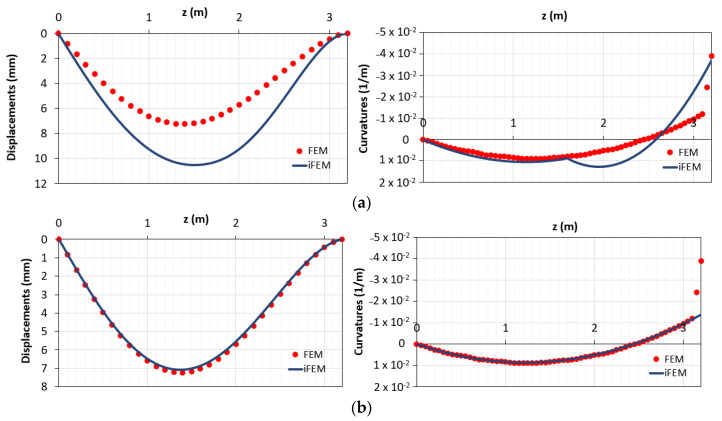
(**a**) Displacements and curvatures diagrams for the configuration “a”; (**b**) displacements and curvatures diagrams for the configuration “b”.

Figure 19 reports the comparison between the displacements and curvatures diagrams, obtained with FEM and iFEM method, for the configurations “a” and “b”.

It can be seen that the displacements of the configuration “b” (Figure 19b (left)) are more accurate than the displacements of the configuration “a” (Figure 19a (left)) because, even if the plastic zone is unknown in terms of curvature, it represents only a small contribution on the whole length of the structures. On the other hand, despite the presence of the station point in the plastic zone for the configuration “a”, there is an increase of errors due to the presence of the second element that models both the zones below and above M_y_. All the observations can be interpreted in terms of root-mean-squares of the displacements (e_RMS,d_) and curvatures (e_RMS,c_) (Table 2).

Similar to the statically determinate structure, also, in this case, some requirements are necessary:

- Check of the curvatures to model each zone in a different phase with its inverse element;
- A procedure that allows obtaining the curvatures in the plastic zone if this exhibits a lack of station points.

#### Hybrid Method for Statically Indeterminate Structures

In statically indeterminate structures with nonlinear material behavior, in the presence of an increasing load, a nonlinear response is evidenced, and this determines a redistribution capacity. In case of lack of input data for the plastic area, it is not possible to refer to the procedure described in Section 4.1.1. An alternative method supported by an additional step using a local FE model, also considering the limited length of the plastic area compared to the structures, is proposed below; it is based on the assumption that the plastic zone is located in a small portion of the beam and this is widely supported by actual evidence in reinforced concrete structures:

1. Perform the iFEM analysis with the input data from the strain sensors (FEM simulated) outside the plasticized areas (checking curvatures lower than yielding curvatures) and identify the exact length of the plastic areas;
2. Estimate the input data in the plasticized areas (auxiliary station points) from a direct local FEM analysis of a reduced statically determinate scheme, having at the edge of the model (beginning/end of the plastic areas) an imposed load conditions given by transverse displacement and rotation predicted by the iFEM analysis;
3. Perform a new iFEM analysis with the initial input data and the estimated value for the plasticized area (auxiliary station points from data simulated by the local FE model).

**Figure 20:**
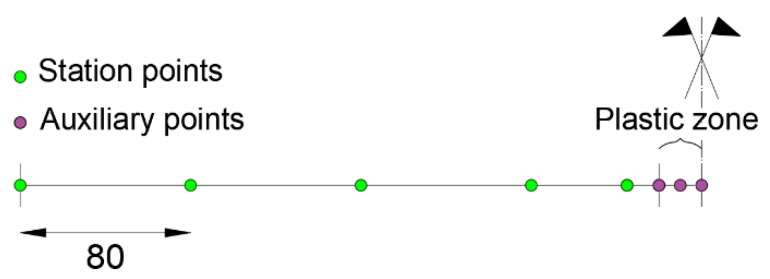
Modeling scheme for the proposed procedure.

In case the initial station points are positioned in the plastic zone, step (2) is not required, but for a more refined analysis, the length of the plasticized zone should be defined in step (1). The procedure should be repeated for every loading step. The results obtained by applying the procedure to configuration “b” related to the case of missing input data in the plastic zone are presented below. In the direct FEM analysis, as a reduced statically determinate scheme, a cantilever beam with a length of 0.4 m (distance from the central support) is considered (step (2)). The iFEM analysis (step (3)) is performed with two inverse elements: one for the elastic zone and one for the plastic zone (Figure 20).

**Figure 21:**
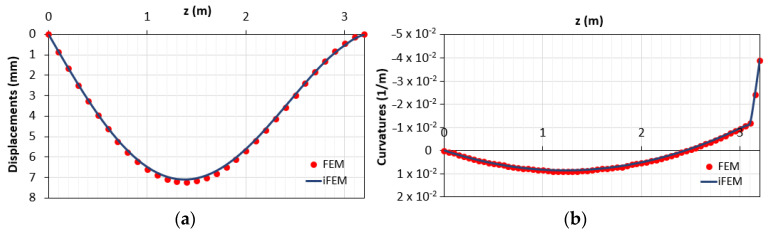
Results obtained with the proposed method: (**a**) displacements diagram; (**b**) curvatures diagram.

The final diagrams of the displacements and curvatures obtained after performing step (3) are shown in Figure 21.

**Figure 22:**
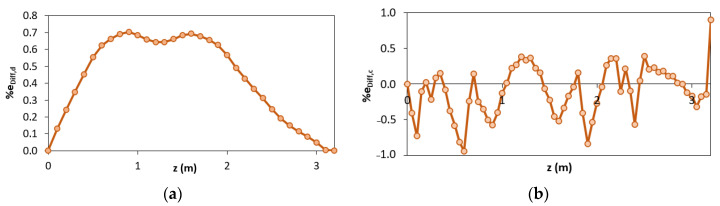
Percentage differences related to: (**a**) displacements (e_Diff,d_); (**b**) curvatures (e_Diff,c_).

The previous analysis provides the results with the percentage of differences reported in Figure 22.

It can be seen that, for the present level of loading, the maximum percentage difference is less than 1% for both the displacements and the curvatures. The curvature root-mean-square of the configuration “b” decreases from e_RMS,c_ = 8.80% to e_RMS,c_ = 0.36%.

## 5. Conclusions

In the present paper, displacement and strain field reconstruction of reinforced concrete beams affected by material nonlinearity is addressed by using the iFEM algorithm. The iFEM formulation originally developed by Tessler and Spangler [7] is based upon the minimization, in a least-square sense, of a functional error between analytical and discrete experimental strains measured by strain sensors. Several inverse models have been developed so far, including plate, shell, Timoshenko and Bernoulli–Euler beam theory, mainly tested in aeronautical and marine structures. To the best knowledge of the Authors, this study applies, for the first time in the open literature, iFEM in the presence of material nonlinearity deriving from the structural behavior of concrete structures in civil engineering. For this purpose, model configurations for statically determinate and indeterminate reinforced concrete beams are analyzed. Unlike the cases with linear elastic material behavior where the deformations are represented by continuous functions, for elements with nonlinear behavior, the knowledge of the mechanical properties is requested. A further critical issue arises in the case of the lack of input data in the plastic zone. Hence, two hybrid methods have been proposed to obtain the input data in such locations, named “auxiliary points”. The nonlinear FEM analyses are used to compute the simulated sensor strains. The comparisons between the reference solutions obtained from this nonlinear FE model and the structural responses reconstructed from iFEM (supported by bending moment-curvature relationship) demonstrate that the iFEM algorithm is a promising system also for structures characterized by material nonlinearities such as bridge structures. Future efforts will be devoted to the execution of experimental tests on full-scale reinforced concrete beams in order to test the accuracy of the results with real noisy strain measurements. The outcomes of the present work can be directly used for bridge monitoring by introducing constitutive equations and then performing a real-time damage prediction other than comparing the reconstructed deformed shape and alert thresholds.

## Figures and Tables

**Table 1:** Root-mean squares of the displacements and curvatures.

Configurations	e_RMS,d_ (%)	e_RMS,c_ (%)
a	14.76	16.82
b	12.67	14.59
c	1.72	6.32
d	9.21	8.77

**Table 2:** Root-mean-squares of the displacements and curvatures.

Configurations	e_RMS,d_ (%)	e_RMS,c_ (%)
a	33.03	13.45
b	1.62	8.80

## Data Availability

Data is contained within the article.
